# A Tragic Case of Orbital Cellulitis in an Immunocompromised Patient

**DOI:** 10.7759/cureus.93857

**Published:** 2025-10-05

**Authors:** Chin Ling Tan, Nur Afzan Mohd Jaffar

**Affiliations:** 1 Ophthalmology, Sarawak General Hospital, Kuching, MYS

**Keywords:** hiv/aids-related opportunistic infections, immunocompromised, multiorgan involvement, orbital cellulitis, orbital manifestations

## Abstract

Immunocompromised patients are prone to opportunistic infections. Orbital infections are most likely to occur in advanced stages of acquired immunodeficiency syndrome (AIDS) and are associated with a high mortality rate, making prompt diagnosis and intervention critical. This case report describes a 42-year-old man, previously without known medical conditions, who presented to the Ophthalmology Department with a two-day history of swelling in his right eye. Additional symptoms included a productive cough with greenish sputum for two weeks and constitutional symptoms for one week. Examination revealed features of right orbital cellulitis and diminished optic nerve function. In addition, he had multiple tattoos all over his body with numerous pustules appearing on the forehead and lower limbs. He was clinically septic with rapidly deteriorating vital signs. Laboratory findings indicated a low total white cell count and neutropenia. Imaging studies disclosed lung and orbital abnormalities, and a rapid human immunodeficiency virus (HIV) test yielded a positive result. Despite aggressive treatment, the patient's clinical status rapidly declined, culminating in multiorgan failure and fatality. This case highlights a prompt recognition of orbital cellulitis in immunocompromised patients, which is imperative, as it may signify disseminated HIV infection and carry substantial morbidity and mortality risks.

## Introduction

Human immunodeficiency virus (HIV) and acquired immunodeficiency syndrome (AIDS) are major public health issues globally. According to the latest updates from the World Health Organization (WHO), approximately 40.8 million people were living with HIV at the end of 2024 globally. Of these, there were approximately 630,000 deaths. In 2024 alone, 1.3 million people were diagnosed with HIV [1].

Orbital manifestations in AIDS patients are infrequent [2-5]. The most common presentations include orbital cellulitis, Kaposi sarcoma, and non-Hodgkin lymphoma [3]. The prevalence of these conditions is highly dependent on the CD4 count of patients. Orbital infections are most likely to occur in advanced stages of AIDS and are associated with a high mortality rate [2].

## Case presentation

**Figure 1:**
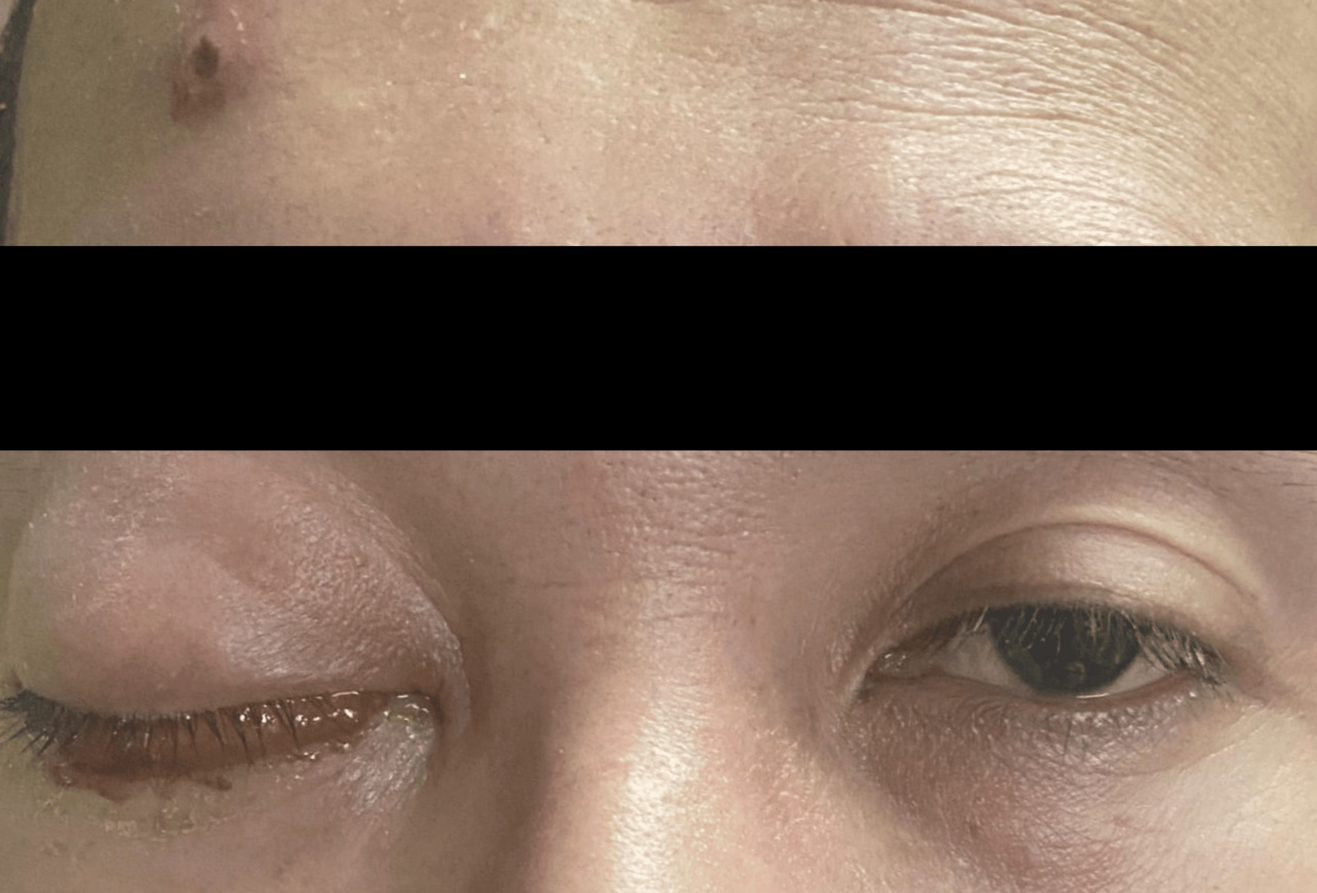
Right eye periorbital swelling with proptosis and skin pustule over the forehead

A 42-year-old man, previously without known medical conditions, presented to the Ophthalmology Department of Sarawak General Hospital with a two-day history of swelling in his right eye (RE) (Figure 1). Additional symptoms included a productive cough with greenish sputum for two weeks and constitutional symptoms such as loss of weight and loss of appetite for a week.

**Figure 2:**
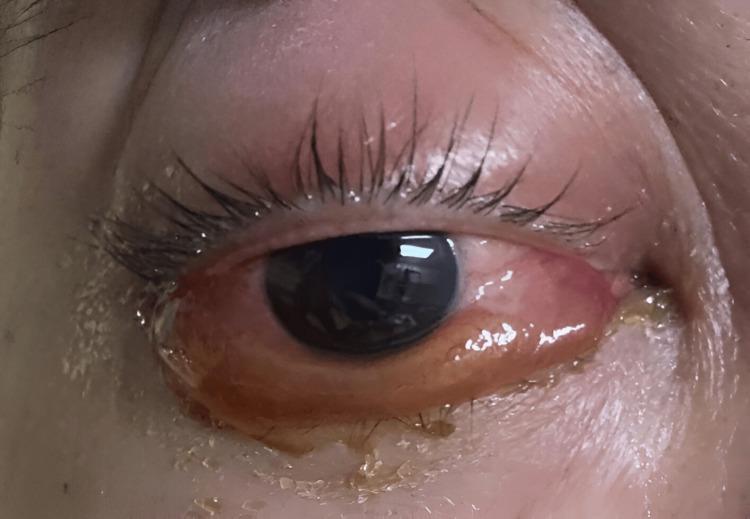
Right eye generalized chemosis which was more prominent inferiorly

The presenting visual acuity of the RE was 6/18 and 6/12 with pinhole, while that of the left eye (LE) was 6/9. RE had relative afferent pupillary defect. The anterior segment of the RE showed erythematous and swollen lid and injected conjunctiva with generalized chemosis, which was more prominent inferiorly (Figure 2). There was no anterior chamber cellular activity. The anterior segment of the LE was unremarkable. Intraocular pressure of the RE was raised (32 mmHg), whereas it was normal over the LE. The Ishihara test revealed a color defect over the RE. RE extraocular movement was limited at all gazes, while it was normal over the LE. Fundus examination of both eyes was unremarkable. There was no optic disc swelling or choroidal folds detected. The diagnosis of right orbital cellulitis was made in keeping with these clinical ocular features.

**Figure 3:**
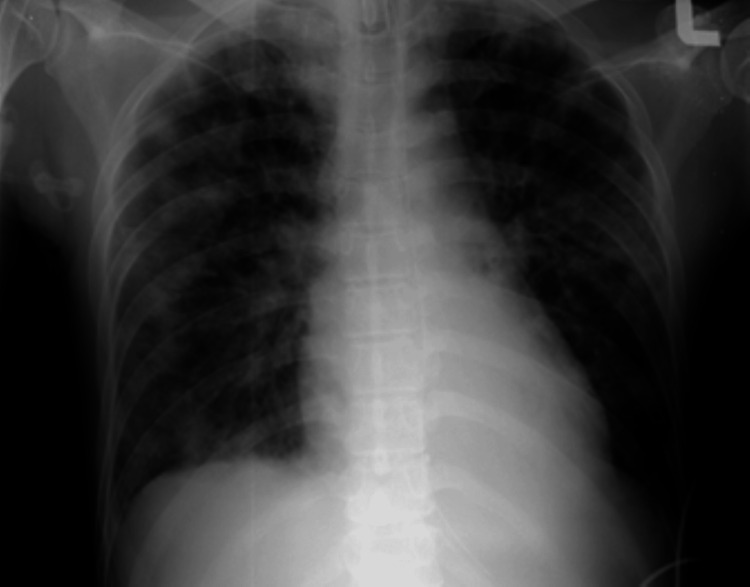
Chest X-ray showing generalized ground-glass opacities over both lung fields

Systemic examination revealed multiple tattoos all over his body with numerous pustules on the forehead (Figure 1) and lower limbs. There were whitish patches on the tongue. Lung auscultation revealed coarse crepitus over the bilateral midzone. The chest X-ray (CXR) showed generalized ground-glass opacities (Figure 3).

The patient was found to be tachypneic and tachycardic the next day. He was immediately referred to the medical team in view of rapidly declining clinical signs and suspicion of a systemic immunocompromised state. Despite these alarming signs, the patient was not treated adequately.

Laboratory findings indicated a low total white cell count of 0.57 (10^3^/µL), neutropenia, and lymphopenia, undoubtedly confirming the immunocompromised state. A rapid HIV test yielded a positive result. He was subsequently referred to the infectious disease team. The diagnosis was revised to severe HIV infections with multiple organ involvement, including orbital cellulitis, pneumonia, oral candidiasis, and skin infections. He was then commenced on intravenous piperacillin/tazobactam, cloxacillin, and fluconazole to treat the polymicrobial infections.

**Figure 4:**
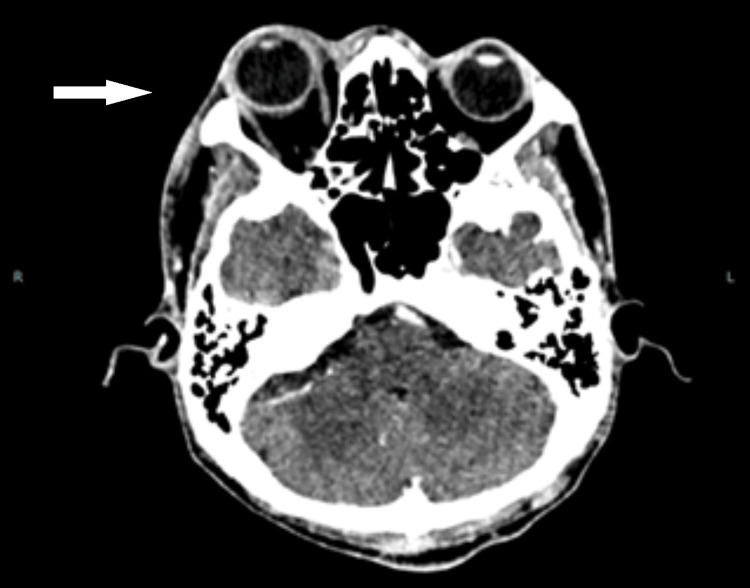
Thickening and enhancement of the right globe wall with pre-scleral fat streakiness. Right eye proptosis. Ethmoid sinuses were clear

Contrast-enhanced computed tomography (CECT) of the brain and orbit during the initial presentation showed thickening and enhancement of the right globe wall with pre-scleral fat streakiness. The fat stranding was also observed in the pre- and post-septal regions and the retroorbital fat, all of which were suggestive of right orbital cellulitis and posterior scleritis. There was minimal mucosal thickening over the bilateral ethmoid and maxillary sinuses (Figure 4). There were no focal brain lesions or features of cavernous sinus thrombosis.

Nasal endoscopy by the otolaryngologist (ear, nose, and throat (ENT)) team revealed clear sinuses, ruling out sinusitis as the cause of orbital cellulitis.

Subsequent investigations revealed a CD4 count of 6 cells/µL, and syphilis serology was positive. Other infectious screenings, such as cytomegalovirus polymerase chain reaction (CMV PCR), hepatitis B, and hepatitis C, were negative. Blood culture grew *Staphylococcus aureus*. Serum cryptococcal antigen was positive.

Despite aggressive treatment, the patient's clinical status rapidly declined. Eventually, he succumbed to multiorgan failure.

## Discussion

AIDS patients are prone to opportunistic infections, which frequently involve the anterior and posterior segments of the eye. They rarely involve the orbit [2]. A study by Moraes found that around 25% of untreated HIV patients presented with adnexal and orbital manifestations [3]. Another study by Hothi et al. showed that the prevalence of ocular manifestations in HIV patients receiving highly active antiretroviral therapy (HAART) was 39%. Among these patients, 20% had adnexal involvement, 28% anterior segment involvement, 33% posterior segment involvement, 11% neuro-ophthalmic abnormalities, and 4% orbital involvement [4]. Orbital cellulitis accounts for less than 1% of the cases, which is a rare condition [3,5].

The commonest cause of orbital cellulitis in immunocompetent patients is bacterial sinusitis, which often originates from the ethmoid and maxillary sinuses [2]. Other predisposing factors include recent facial trauma, a history of dental procedures, and hematogenous spread from opportunistic infections [5,6]. Common causative organisms include *Staphylococcus aureus* (50%) [5], *Streptococcus pyogenes*, and *Streptococcus pneumoniae*. Others include fungi such as *Aspergillus* and mycobacteria [2,5]. Based on a case series by Mansour, *Staphylococcus aureus* infection is twice as common in HIV patients as compared to healthy individuals [7]. This patient most likely acquired orbital cellulitis through hematogenous spread from *Staphylococcus aureus* infection, evidenced by a positive blood culture. Sinusitis was ruled out by the nasal endoscope.

According to Meyer and Smit, polymicrobial infections are present in 38.1% of cases, often spreading via the hematogenous route (25.4%) [5]. Half of the patients with orbital cellulitis died within one year of the infection, usually due to invasive *Aspergillus fumigatus* infection with intracranial extension [2,5]. They typically have a low CD4 lymphocyte count, which is less than 100 cells/mm³, and are often associated with neutropenia [2,5]. This patient had a very low CD4 count with neutropenia. He was suspected of having polymicrobial infections in view of his severe immunocompromised state.

Broad-spectrum antibiotics should be considered, including antibacterials, such as vancomycin, ceftriaxone, cefotaxime, ampicillin-sulbactam, piperacillin-tazobactam, and metronidazole, and anti-fungal, such as fluconazole [5,8]. This patient was given intravenous piperacillin/tazobactam, cloxacillin, and fluconazole. Clinically, the orbital cellulitis responded well to the antibiotic regimes, and the chemosis and proptosis resolved by the second day of treatment. 

## Conclusions

Orbital cellulitis is a rare presentation of opportunistic infection in immunocompromised patients. Prompt recognition of orbital cellulitis in immunocompromised patients is imperative, as it may signify disseminated HIV infection and carry substantial morbidity and mortality risks. This case underscores the importance of early and appropriate intervention.
